# Induction with Rabbit Antithymocyte Globulin following Orthotopic Liver Transplantation for Hepatitis C

**Published:** 2011-11-01

**Authors:** R. F. Saidi, M. Hertl, R. T. Chung, D. S. C. Ko, T. Kawai, J. Markmann, A. K. Bhan, A. B. Cosimi, N. Elias

**Affiliations:** 1 *Department of Surgery-Transplantation Unit, *; 2 *Gastrointestinal Unit, Department of Medicine, *; 3 *Department of Pathology, Massachusetts General Hospital, Harvard Medical School, Boston, MA, USA, *

**Keywords:** Liver transplantation, Hepatitis C, Induction, Recurrence

## Abstract

Background: Hepatitis C (HCV) is the most common indication for liver transplantation in the US.

Objective: Since steroids are the major stimulus of viral replication, we postulated that steroid-free immunosuppression might be a safer approach.

Methods: From January 1995 to October 2002, we used steroid plus calcineurin inhibitor (CNI) immunosuppression after liver transplantation for HCV (steroid group, n=81). From October 2002 to June 2007, rabbit antithymocyte globulin (RATG) induction, followed by CNI and azathioprine (RATG group, n=73) was utilized.

Results: There were no differences in 1- and 3-year patient/allograft survival rates. The incidence of acute rejection rate (19% *vs*. 28%), of biopsy-proven HCV recurrence (70% *vs*. 75%), and chronic rejection (6% *vs*. 9%) were comparable. The mean time to develop recurrent HCV was significantly longer in the RATG group (16.2 *vs*. 9.2 months, p=0.008). The incidence of severe portal fibrosis appears to be lower in RATG group compared to the steroid group; 14% *vs.* 4% (p=0.07).

Conclusions: RATG induction is safe and effective after liver transplantation for HCV, but has no impact on the incidence of HCV recurrence and patient/allograft survival. However, a significant delay in time to HCV recurrence and a trend toward less rejection and portal fibrosis was observed.

## INTRODUCTION

Hepatitis C (HCV) is the most common indication for liver transplantation in the US, accounting for 40%–45% of all liver transplants [1-3]. Unfortunately, disease recurrence is universal in patients who are viremic before transplantation and leads to cirrhosis in at least 25% of patients five years after liver transplantation. This discouraging outcome makes HCV an important but still contentious indication for retransplantation [1-3].

The course of recurrent HCV is variable and several factors, including the type of immunosuppression used, have been proposed to be associated with early and more severe recurrent HCV. Use of steroid-free immunosuppression following liver transplantation has gained attention in recent years due to the potential for fewer complications such as diabetes and osteopenia and also for hopefully impacting favorably the course of recurrent HCV [4-6]. Antilymphocyte antibody induction is also increasingly utilized because it minimizes the need for calcineurin inhibitors (CNI) in the immediate post-operative phase, hence potentially limiting the renal toxicity in patients with preexisting renal insufficiency and also reduces the need for high-risk steroid treatment of acute rejection [6]. In this study, we analyzed the outcome of patients who underwent liver transplantation for HCV comparing rabbit antithymocyte globulin (RATG) induction *vs*. steroid induction and maintenance.

## MATERIAL AND METHODS

We analyzed the outcome of liver transplantation for HCV in a cohort of 154 consecutive patients. From January 1995 to October 2002, we used a CNI/steroid immunosuppressive protocol after liver transplantation for HCV (steroid group, n=81). From October 2002 to June 2007, we administered induction with RATG, a CNI and azathioprine (RATG group, n=73). The RATG group patients received 1.5 mg/kg of rabbit thymoglobulin begun during the anhepatic phase followed by a repeat dose on days one, two and three. Patients were given 650 mg of acetaminophen and 50 mg of diphenhydramine orally before each RATG infusion. The recipients in the steroid group received 250 mg of methylprednisolone during the anhepatic phase and were tapered from 200 mg/day to 20 mg of prednisone by day six followed by low dose oral steroids (2.5–5 mg once daily by three months).

All patients received surgical site prophylaxis with a first-generation cephalosporin for 24 hours, antifungal prophylaxis with clotrimazole for five days, and anti-Pneumocystis prophylaxis with sulfamethoxazole/trimethoprim indefinitely. The prophylaxis regimen was tailored if there was a history of allergy to standard protocol drugs. Antiviral prophylaxis consisted of oral valganciclovir for 4–6 months, if either donor or recipient cytomegalovirus (CMV) serologic status was positive. Oral famciclovir was used if both donor and recipient serologies were negative. In the event of donor CMV positivity and recipient CMV negativity, the antiviral prophylaxis continued out to one year and CMV antigenemia assay was used to detect subclinical replication that might dictate length of prophylaxis therapy. Before valganciclovir become commercially available (June 2001), oral ganciclovir 1000 mg PO, TID was used.

Allograft biopsy was triggered by abnormal liver function tests beyond expected post-operative period with or without HCV viremia. In addition, 27 patients in RATG group underwent one-year protocol biopsy. Histological confirmation of HCV recurrence was defined in liver biopsy specimens as described by Ishak and collegues [7]. The time to recurrence was defined as “early:” from day 0 to day 100; “intermediate:” from day 101 to day 180; and “late:” from day 181 to day 395.

Patient and graft survival, rejection, incidence of infectious complications, and the incidence and severity of recurrent HCV were evaluated in both groups. The mean follow-up was 48 (range: 17–120) months. Statistical comparisons of the steroid and RATG patients were performed using *Student’s t* test, χ^2^ test and Kaplan-Meier survival analysis. A p value <0.05 was considered statistically significant.

## RESULTS

**Figure 1:**
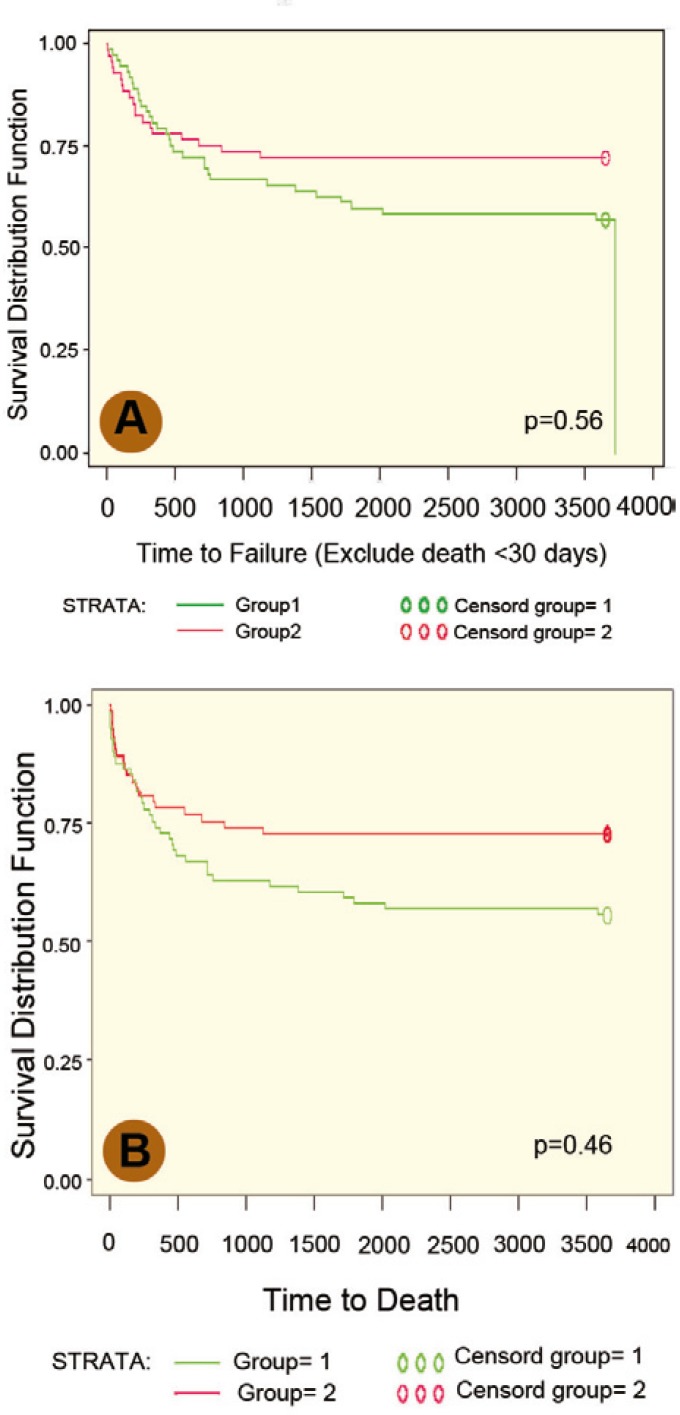
Kaplan-Meier analysis showed comparable allograft (A) and patient survival (B) in both groups. Group I is steroid group and Group II received RATG

Table 1 confirms that the demographic and clinical characteristics of donors and recipients in both groups were comparable. Overall, 30-day peri-operative mortality was 14/154 (9.1%): five (6%) in RATG group and nine (11%) in steroid group (p=0.9). In the RATG group, two patients died of primary nonfunction (PNF) and three died of sepsis. In the steroid group, the causes of death were PNF in one, sepsis in four, cerebrovascular event in three and pulmonary embolism in one. Overall, infections were the most common cause of patients’ mortality in both groups (36% *vs*. 42%). The mean follow-up of the remaining 139 patients was 48 months. Kaplan-Meier survival plots of the two groups (Figure 1) showed no significant difference in allograft and patient survival between the groups.

**Table 1:** Demographic and clinical characteristics of donors and recipients in both groups

	RATG Group (n=73)	Steroid Group (n=81)	p
Age	49.6±8	51.7±6	NS*
Male gender (n, %)	47, (64.4%)	56, (69.1%)	NS
MELD (calculated)	29±7	27±6	NS
HCV genotype I	76%	74%	NS
Donor age	46±6	42±8	NS
Donor sex (M/F)	38/35	42/39	NS
Donor cause of death			NS
Trauma	38%	45%	
CVA**	45%	42%	
Anoxic	12%	7%	
Other	5%	6%	
Donor BMI	27.6±5	25.4±7	NS
Cold ischemic time (hour)	8.1±1.8	7.9±2.1	NS
Warm ischemic time (minutes)	40±6	42±7	NS

* NS: Not significant

** CVA: Cerebrovascular accident

The incidence of bacterial or fungal infectious complications requiring hospital management was not different between the two groups (54.8% in the RATG group *vs*. 39.5% in the steroid group, p=0.72). The incidence of multiple re-admissions for infectious complications was 16.1% and 17.8% in both groups, respectively (p=0.85).

**Figure 2:**
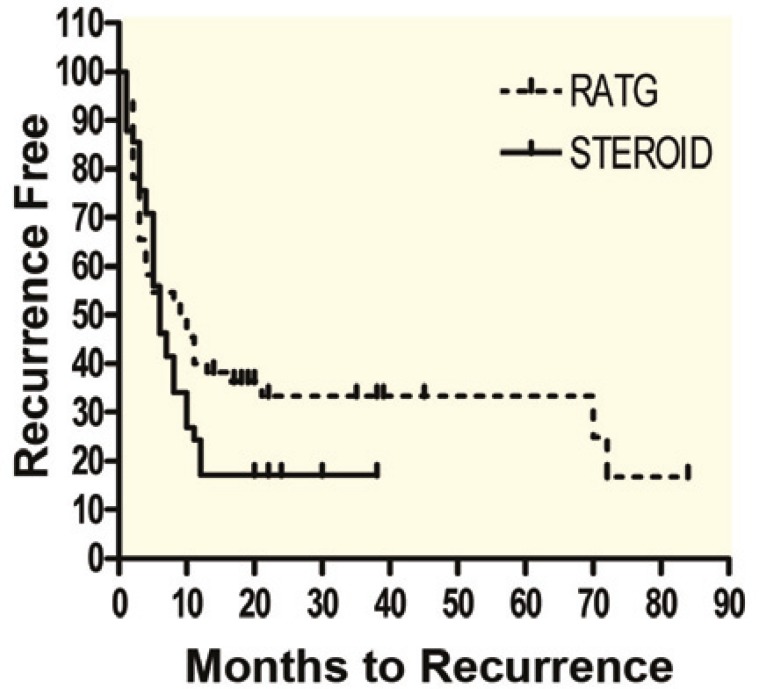
Time (months) to development of recurrent hepatitis C was longer in RATG group (p=0.008).

The ultimate incidence of biopsy-proven HCV hepatitis recurrence was comparable in both groups (70% *vs*. 75%, respectively). As shown in Figure 2, The mean±SD time to HCV recurrence was 16.2±2.7 (95% confidence interval [CI]: 10–21) months in the RATG group compared to 9.2±1.3 (95% CI: 6–12) months in the steroid group (p=0.008). Most strikingly, the incidence of early HCV hepatitis recurrence (within three months post-transplant) was 9.2% in the RATG group compared to 29.6% in the steroid group (p<0.001).

The overall acute rejection rate was 28% in the steroid group *vs*. 19% in the RATG group (p=0.72). The incidence of chronic rejection (6% *vs*. 9%) and fibrosing cholestatic hepatitis (4% *vs*. 1%) was comparable in both groups. The incidence of portal fibrosis (stage 2 or greater) was lower in the RATG group compared to the steroid group (14% *vs*. 4) but did not reach a statistical significance (p=0.07).

## DISCUSSION

Chronic infection with HCV is a frequent cause of cirrhosis and hepatocellular carcinoma worldwide, and has become the most common indication for liver transplantation in the US, accounting for approximately 40%–45% of all liver transplants [1-3]. Unfortunately, recurrent HCV is a universal event with serious consequences for many recipients including cirrhosis and liver failure. Recurrent HCV leads to cirrhosis in 10% to 25% of transplant recipients within 5 to 10 years [1-3].

Several donor, recipient, and viral factors correlate with HCV disease severity following transplantation. Some studies have suggested that recipients of living donor grafts and those with high pre- or post-transplantation viral load may be more likely to develop severe recurrent disease [2-4]. Patients infected with genotype 1 HCV and those who develop CMV infection are also at increased risk for severe disease recurrence [2]. Immunosuppressive agents, specifically corticosteroids and OKT3, have also been identified as possible risk factors for HCV recurrence [1, 2].

It is well known that liver disease caused by HCV infection progresses more rapidly in immunosuppressed than in immunocompetent individuals [1]. The role of specific immunosuppressive agents on the evolution of post-transplantation HCV disease has been evaluated to some extent, but the results have been inconclusive [1-3]. Whether or not the initial antibody induction, the type of CNI used, the addition of other immunosuppressive agents, or steroid maintenance definitely influence outcome remains a matter of debate. Nevertheless, it is well accepted that acute rejection episodes requiring the increased use of steroids and/or antibodies enhance the risk of HCV recurrence through immunological mechanisms [1, 2]. Therefore, optimal prevention of acute rejection should represent a goal for reducing the rate of graft HCV infection. An optimal immunosuppression regimen should use the fewest agents at the lowest effective doses in order to reduce toxicity and cost, but still prevent rejection and disease recurrence.

Corticisteroids have been the most commonly used immunosuprresive agent after CNI in liver transplant recipients. Unfortunately, acute and chronic administration of corticosteroids is associated with numerous adverse effects including hypertension, hyperglycemia, delayed wound healing, osteoporosis, glaucoma, suppressed growth, hyperlipidemia, increased risk of gastrointestinal ulceration, risk of fungal infections, and suppression of the pituitary-adrenal axis [4-6]. In terms of the impact of specific immunosuppressive agents on long-term outcomes of patients transplanted for HCV, there is little doubt that the use of corticosteroid boluses to treat acute cellular rejection is harmful to HCV-infected recipients. Thus, corticosteroid-free maintenance immunosuppression regimens have been investigated in several relatively small series of HCV^+^ patients. Reported benefits include suppression of HCV RNA levels, reduced incidence of advanced fibrosis, and less severe recurrence of hepatitis C [8-10].

The use of induction therapy in liver transplantation has not been as common as in other solid organ transplantation. Nevertheless, about 25% of liver transplant recipients receive induction therapy [11, 12]. Antibody induction is viewed with caution because it is liable to cause severe side effects and to promote perioperative complications, such as infection.

Antibody-induction therapy has been limited to the peri-operative period as a means of reducing early exposure to CNI or to obviate the need for large doses of peri-operative corticosteroids. RATG, a polyclonal antilymphocyte antibody preparation, is the most commonly used induction antibody in the US. RATG has been used as an induction agent in liver transplantation to reduce or eliminate corticosteroids use, minimize the use of CNI, delay exposure to CNI in patients with pre-existing renal failure, and explore the possibility of eliminating maintenance immunosuppression [11-17].

The use of RATG as part of a steroid-free protocol gained increasing popularity when an early randomized controlled trial suggested a reduced incidence of recurrent HCV in RATG-treated patients (50%) *vs*. steroid bolus recipients (71%), although this difference was not statistically significant [4]. These encouraging findings led to widespread use of RATG and as of September 2007, several centers have reported their experience with RATG without steroids *vs*. steroid induction for recurrent HCV [11-17]. The results were conflicting, with several centers reporting a higher incidence of acute rejection in non-RATG patients and others stating RATG had no impact on graft or patient survival. 

Eason and colleagues [13] randomized 119 patients after liver transplantation to receive RATG 1.5 mg/kg on day 0 and 1, *vs*. methylprednisone 1 g followed by a steroid taper over three months, combined with maintenance treatment of tacrolimus and MMF for three months. Patient and graft survival and incidence of acute cellular rejection (ACR) were similar in both groups. Patients in the corticosteroid group had more severe ACR, which required additional steroid treatment (50% of patients with ACR compared with 7% in the RATG group). Although the study was not designed to evaluate specifically the treatment of HCV recipients, analysis of this subset of patients revealed that thymoglobulin induction was also associated with a reduced incidence of recurrent HCV. Other advantages included less post-transplantation diabetes mellitus, and CMV infection, with no increased risk of overall infectious complications in the RATG group. In our study, the incidence of recurrent HCV was comparable in both groups. However, the patients who received RATG had a significant delay in time to develop recurrent disease (16.2 months) compared to those who received steroid induction and maintenance (9.2 months). In addition, the RATG and steroid groups had comparable rejection rates (19% *vs*. 28%) but a trend toward less portal fibrosis (4% *vs*. 14%). The fact that the RATG delayed recurrent hepatitis C following liver transplant could be related to steroid avoidance or RATG itself. It was recently been shown that RATG-mediated immunosuppression is delivered in part via immunologically specific actions involving the generation of regulatory T cells (Treg), particularly CD4^+^ CD25^high^ Foxp3^+^ cells. This is mainly due to RATG unique ability to convert the CD4^+^ CD25^–^ T cells into CD4^+^ CD25^+^ T cells.^18^ Tregs play a central role in viral persistence and immunologic reaction against HCV infection [19, 20].

Tector and colleagues [16] at Indiana University used RATG induction combined with delayed low-dose CNI treatment. They demonstrated a one-year graft and patient survival of 92% and 96%, respectively, combined with a low incidence of ACR (6%) and a reduced incidence of renal complications. The most common adverse effects were fever, rigors, and tachycardia. Our study also showed comparable patient and allograft survival comparing RATG with steroids. Interestingly, our patients who received RATG developed less severe portal fibrosis (4% *vs*. 14%) compared to steroid group which was not statistically significant and may need longer follow-up.

This study has several limitations. First, it is a retrospective analysis with relatively small sample size. Second, the follow-up was longer in the steroid group compare to the RATG group. Third, protocol biopsies were not done routinely.

In conclusion, our study showed that RATG induction therapy has similar results compared to steroid regimen in terms of long-term patient and allograft survival, recurrent hepatitis C, and acute rejection. However, RATG delayed the time to recurrence and appeared to lessen the rate of progression as there was a trend toward lower incidence of portal fibrosis in the patients who received antibody induction. These encouraging findings warrant further study and approach longer follow-up.
